# Supplementary material for: Temperature-Resistant Intrinsic High Dielectric Constant Polyimides: More Flexibility of the Dipoles, Larger Permittivity of the Materials
Source: Molecules. 2022 Sep 26;27(19):6337. doi: 10.3390/molecules27196337 (PMC9571362; doi:10.3390/molecules27196337)
Supplement: Supplementary file 1 [file molecules-27-06337-s001.zip › molecules-1926138-supplementary.pdf]

Supporting Information

Temperature Resistant Intrinsic High Dielectric Constant Polyimides: More Flexibility of the Dipoles, Larger Permittivity of the Materials

Weiwen Zheng[§], Zuhao Li[§], Kaijin, Chen, Siwei Liu, Zhenguo Chi, Jiarui Xu and Yi Zhang*

1. Theoretical simulations 2

2. Results and discussion

Figure S1. The FT-TR spectrum of C0-SPI, C1-SPI, and C2-SPI 2

Figure S2. The XRD curves of C0-SPI, C1-SPI, and C2-SPI. 2

Table S1. Mechanical properties of C0-SPI, C1-SPI, and C2-SPI 3

Figure S3. Eyring equation plot of -lnf_{peak} versus 1/T for the frequency–scan BDS results of (**a**) C0-SPI, (**b**) C1-SPI, and (**c**) C2-SPI. 3

Table S2. RMSF of each fragment on the side chains of C0-SPI, C1-SPI, and C2-SPI (unit： nm) 3

Figure S4. The stacking illustration of the samples of (**a**) C0-SPI, (**b**) C1-SPI and (**c**) C2-SPI. The interval of each frame is 1 ns. 4

Table S3. Variation of the dipole moment before and after applying the external electric field 4

3. Synthesis of monomers and polyimides

Scheme S1. Synthetic routes of the polyimide C0–SPI 4

Scheme S2. Synthetic routes of the polyimide C1–SPI 5

Scheme S3. Synthetic routes of the polyimide C2–SPI 6

1. Theoretical simulations

Quantum mechanics simulation. Quantum mechanics calculation was simulated by Density Function Theory (DFT) in Gaussian 16 program. B3LYP-D3 (BJ) as a hybrid functional and 6-31g (d, p) as a basic set were used for optimization and frequency jobs. The reasonable results are confirmed in VMD 1.9.3. According to the optimization results, M062X and def2-TZVPP were used for energy calculation. B3LYP-D3 (BJ) and TZVP were used for dipole moment and partial atomic charges calculation, and PBE0 and ZPOL were used for optical frequency polarization calculation. The conformation search was carried out by molclus 1.9.9.5, and the criteria for different conformations were geometric deviation greater than 0.1 or energy deviation greater than 0.1 kcal/mol. The average polarizability of the repetitive units was the mean of the results of the corresponding three-unit oligomers.

Molecular dynamics (MD) simulation. MD simulation was performed on gromacs 2021.5. A periodic simulation cell of 30 monomers was employed, and there were 10 polymer chains in the cell. The input files were generated by Sobtop1.0 (Dev2). Restrained electrostatic potential 2 (RESP2) charge with δ values 0.5 and generalized amber force field (GAFF) were used to simulate. The partial atomic charges were obtained from the repetitive units of the polymers by DFT. The simulated cell was periodically annealed at 0-800 K until the internal energy, volume, density, and other parameters of the system stabilized. The NPT ensemble was used in annealing. When the square-mean-root deviation (RMSD) of the system at 298.15 K stabilized within the sampling time, the simulation cells were constructed successfully. The physical properties of the simulation system were sampled for 200 ns at equilibrium.

2. Results and discussion

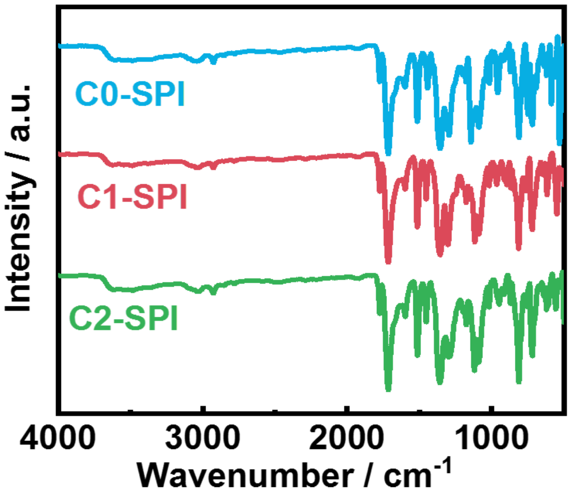

Figure S1. The FT-TR spectrum of C0-SPI, C1-SPI, and C2-SPI.

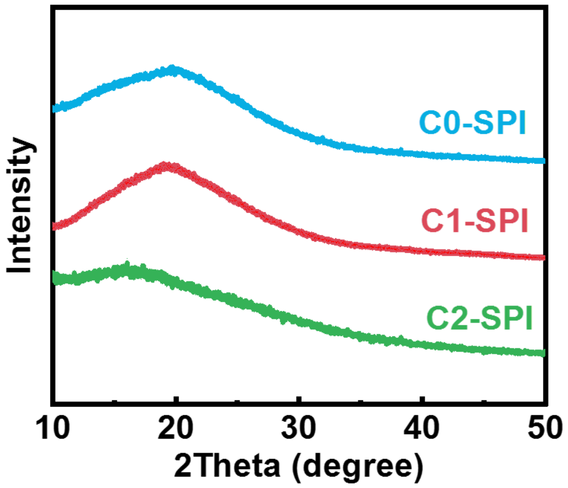

Figure S2. The XRD curves of C0-SPI, C1-SPI, and C2-SPI.

Table S1. Mechanical properties of C0-SPI, C1-SPI and C2-SPI

	C0-SPI	C1-SPI	C2-SPI
Tensile strength / MPa	77.0±4.2	119.2±1.9	144.7±5.5
Elongation at break / %	3.3±0.9	6.4±0.5	12.1±1.1
Tensile modulus / GPa	3.2±0.4	3.3±0.4	2.9±0.3

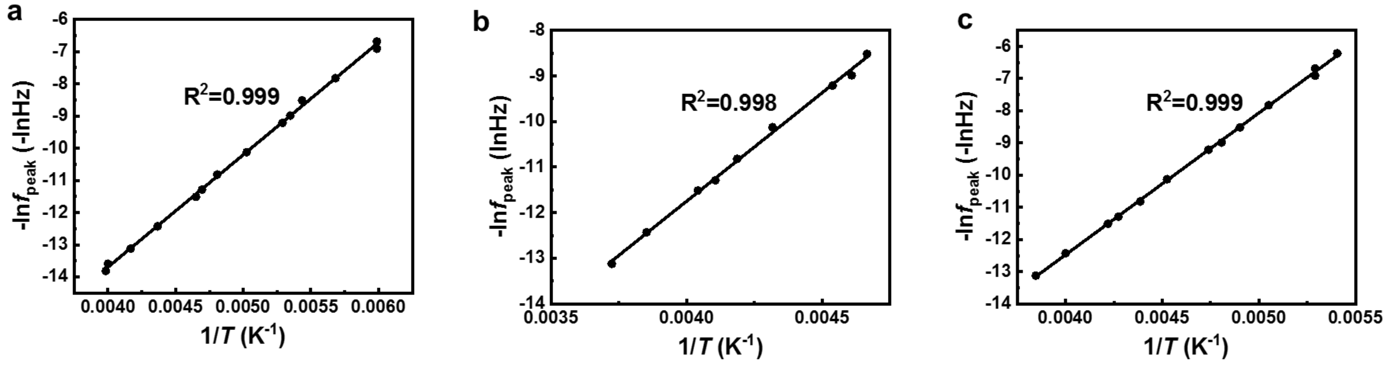

Figure S3. Eyring equation plot of -lnf_{peak} versus $1/T$ for the frequency–scan BDS results of (**a**) C0-SPI, (**b**) C1-SPI, and (**c**) C2-SPI.

Eyring equation fitting. For motion of the side groups in polymers, the relationship between relaxation time and temperature conforms to Eyring equation:

$$\tau = \tau_0 e^{\frac{\Delta E}{RT}}$$

where τ is the relaxation time, τ_0 is the pre-exponential factor, ΔE is the activation energy of relaxation, R is the molar gas constant (8.314 J / mol K), and T is the temperature corresponding to τ. In the frequency–scan BDS measurement, τ is the inverse of the test frequency f_{peak}.

Table S2. RMSF of each fragment on the side chains of C0-SPI, C1-SPI, and C2-SPI (unit： nm)

Groups	$-CH_3$	$-SO_2-$	$-CH_2$-(1)	$-CH_2$-(2)
C0-SPI	0.133	0.0855	-	-
C1-SPI	0.138	0.0904	0.0738	-
C2-SPI	0.148	0.0984	0.0936	0.0782

“-”indicates that molecule does not contain the fragment.

Simulation and calculation of root-mean-square fluctuation (RMSF). RMSF was used to characterize the motility of fragments. RMSF of an atom measures its fluctuation relative to its average position in a simulated trajectory. For atom A:

$$\text{RMSF}_\text{A} = \sqrt{\frac{1}{N}\sum_{i}^{N}(r_\text{A}^i - r_\text{A}^{\text{avg}})^2}$$

where N is the total simulation frame count, r_A^i is the coordinate of atom A in frame i, r_A^{avg} is the average coordinate of atom A. According to the formula, the average RMSF of all atoms in the group is taken as the RMSF of the group to measure its motility.

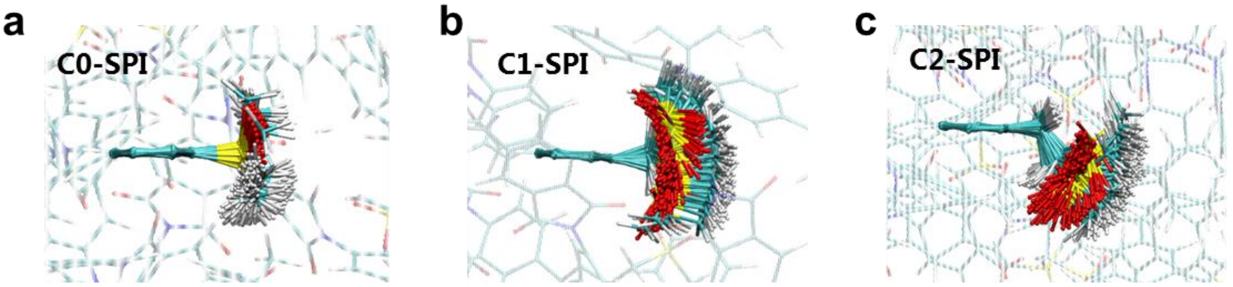

Figure S4. The stacking illustration of the samples of (**a**) C0-SPI, (**b**) C1-SPI, and (**c**) C2-SPI. The interval of each frame is 1 ns.

Table S3. Variation of the dipole moment before and after applying the external electric field

	$\Delta\mu_x$ / D	$\Delta\mu_y$ / D	$\Delta\mu_z$ / D	$\Delta\mu_{avg}$ / D
C0-unit	2.64	0.68	1.42	1.58
C1-unit	2.00	1.57	2.33	1.97
C2-unit	1.88	3.53	3.02	2.81

3. Synthesis of monomers and polyimides

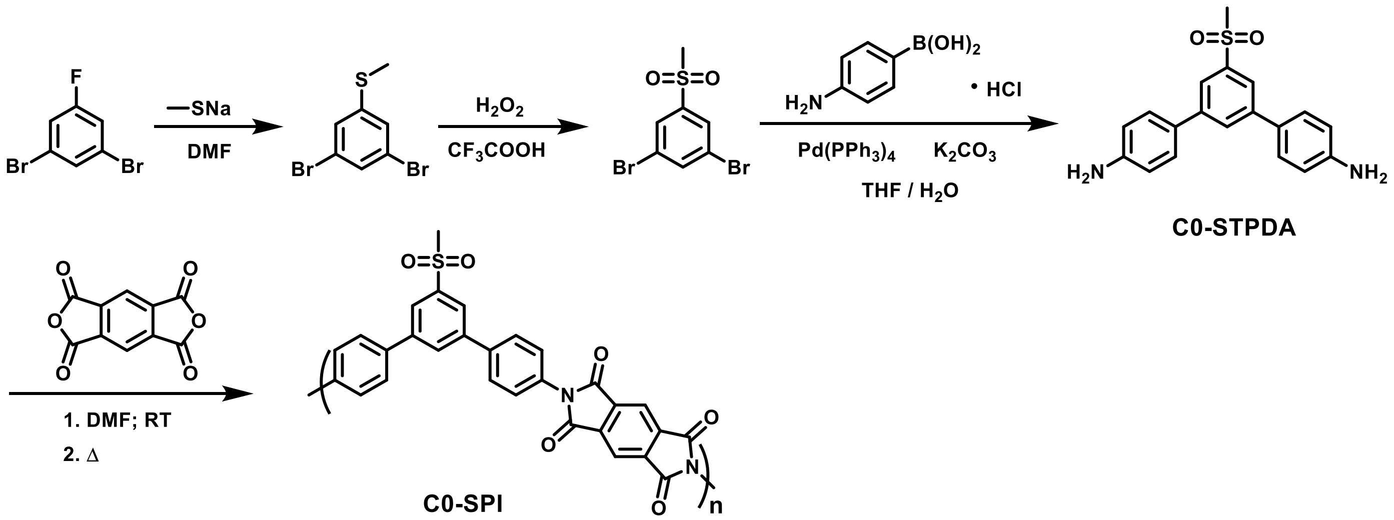

Scheme S1. Synthetic routes of the polyimide C0–SPI.

Synthesis of (3,5-dibromophenyl)(methyl)sulfane. Sodium methanethiolate (2.76 g, 39.39 mmol) was dispersed in DMF (70 mL), and then 1, 3-dibromo-5-fluorobenzene (10 g, 39.39 mmol) was added dropwise at -45 °C. The mixture was stirred at -45 °C for 3 h and at room temperature overnight. The reaction mixture was poured into saturated brine, extracted with ethyl acetate and evaporated under reduced pressure at low temperature to obtain the crude product, which is directly used for the next reaction without further purification.

Synthesis of 1,3-dibromo-5-(methylsulfonyl)benzene. To a stirred solution of crude product in the previous step in 2,2,2-trifluoroacetic acid (23.0 mL) were added 30% H_2O_2 (15.4 mL) at 0 °C, and the reaction mixture was stirred at room temperature for 2 h. After the reaction, 5 M NaOH solution was added until pH=12. The reaction mixture was extracted with ethyl acetate and evaporated under reduced pressure to obtain crude product. The crude product was recrystallized with ethanol to obtain 4.1 g white crystal. ^{1}H NMR (400 MHz, Chloroform-*d*) δ 8.04 - 8.00 (m, 2H), 7.97 – 7.93 (m, 1H), 3.09 (d, J = 1.9 Hz, 3H). ^{13}C NMR (101 MHz, Chloroform-*d*) δ 143.57, 139.31, 129.18, 124.03, 44.48, *m/z*: $[M]^+$ calcd for $C_7H_6Br_2O_2S$, 311.85; found 311.9. Anal. calcd for $C_7H_6Br_2O_2S$: C 26.78, H 1.93; found: C 27.08, H 2.21.

Synthesis of 5'-(methylsulfonyl)-[1,1':3',1''-terphenyl]-4,4''-diamine (C0-STPDA). 1,3-dibromo-5-(methylsulfonyl) benzene (3 g, 9.55 mmol) and $Pd(PPh_3)_4$ (0.22g, 0.19 mmol) were dissolved in THF (50 mL). Then 3 M aqueous K_2CO_3

(25 mL), moderate Aliquat 336 and 4-aminophenylboronic acid hydrochloride (3.98 g, 22.93 mmol) were successively added to the solution, followed by reaction at 70 °C under argon atmosphere for 48 h. The mixture was allowed to cool down to room temperature. After collecting the organic layer by extraction with CH_2Cl_2 and evaporating the solvent, the product was purified by chromatography using CH_2Cl_2 and ethyl acetate as mobile phase to give 1.82 g white powder with a yield of 56.3%. ^{1}H NMR (400 MHz, Chloroform-*d*) δ 7.96 (s, 1H), 7.84 (d, *J* = 1.6 Hz, 2H), 7.53 (d, *J* = 8.2 Hz, 4H), 6.69 (d, *J* = 8.2 Hz, 4H), 5.38 (s, 4H), 3.30 (s, 3H). ^{13}C NMR (101 MHz, Chloroform-*d*) δ 149.72, 142.83, 142.48, 128.17, 127.11, 126.15, 120.90, 114.64, 43.95. *m/z*: $[M]^+$ calcd for $C_{19}H_{18}N_2O_2S$, 338.11; found 338.1. Anal. calcd for $C_{19}H_{18}N_2O_2S$: C 67.43, H 5.36; found: C 67.22, H 5.40.

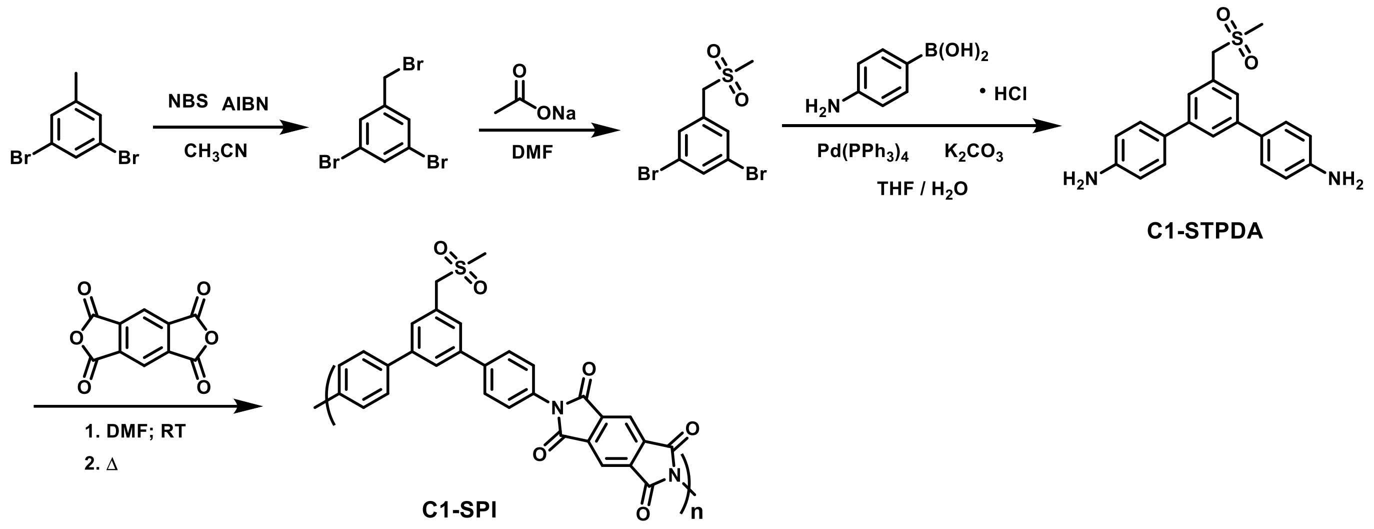

Scheme S2. Synthetic routes of the polyimide C1–SPI

Synthesis of 1,3–dibromo–5–(bromomethyl) benzene. 1,3–Dibromo–5–methylbenzene (15 g, 60.02 mmol), NBS (10.68 g, 60.02 mmol) and AIBN (0.49 g, 3 mmol) were dissolved in 180 ml of acetonitrile, and then the solution was heated to reflux for 3 hours under argon. After removing the solvent under reduced pressure, chromatography with petroleum ether as an eluent gave 10.37 g product as white needle–type crystals with a yield of 53%. ^{1}H NMR (400 MHz, DMSO–d_6) δ 7.80 (t, *J* = 1.7 Hz, 1H), 7.72 (d, *J* = 1.7 Hz, 2H), 4.68 (s, 2H). ^{13}C NMR (101 MHz, DMSO–d_6) δ 142.54, 133.04, 131.09, 122.28, 31.48. Anal. calcd for $C_7H_5Br_3$: C 25.57, H 1.53; found: C 26.07, H 1.55.

Synthesis of 1,3–dibromo–5–((methylsulfonyl) methyl) benzene. 1,3–Dibromo–5–(bromomethyl) benzene (10 g, 30.41 mmol) and sodium methanesulfinate (7.49 g, 73.32 mmol) were added to 75 ml of dry DMF. The mixture was stirred for 3 h at 60 °C under argon, and then poured into 1000 mL of cold saturated NaCl aqueous solution. The white precipitate was collected and dried in a vacuum oven to provide 9.65 g product with a yield of 95.7%. ^{1}H NMR (400 MHz, DMSO–d_6) δ 7.90 (t, *J* = 1.8 Hz, 1H), 7.66 (d, *J* = 1.8 Hz, 2H), 4.55 (s, 2H), 2.96 (s, 3H). ^{13}C NMR (101 MHz, DMSO–d_6) δ 134.14, 133.83, 133.20, 122.80, 58.32. MS (APCI) *m/z*: $[M + H]^+$ calcd for $C_8H_8Br_2O_2S$, 326.86; found 326.9. Anal. calcd for $C_8H_8Br_2O_2S$: C 29.29, H 2.46; found: C 29.69, H 2.55.

Synthesis of 5'–((methylsulfonyl)methyl)–[1,1':3',1''–terphenyl]–4,4''–diamine (C1-STPDA). 1,3–Dibromo–5–((methylsulfonyl) methyl) benzene (5 g, 15.24 mmol) and $Pd(PPh_3)_4$ were dissolved in 90 ml of THF. Then 2 M aqueous K_2CO_3 solution, moderate Aliquat 336 and 4-aminophenylboronic acid hydrochloride were added to the solution, followed by reaction at 70 °C under argon for 24 h. After collecting the organic layer by extraction with CH_2Cl_2 and evaporating the solvent, the product was purified by chromatography using CH_2Cl_2 and ethyl acetate as mobile phase to give 4.1 g white powder with a yield of 77%. ^{1}H NMR (400 MHz, DMSO–d_6) δ 7.65 (t, *J* = 1.7 Hz, 1H), 7.48 – 7.35 (m, 6H), 6.73 – 6.60 (m, 4H), 5.26 (s, 4H), 4.52 (s, 2H), 2.94 (s, 3H). ^{13}C NMR (101 MHz, DMSO–d_6) δ 149.06, 141.85, 130.38, 127.84, 127.59, 125.94, 122.90, 114.64, 67.49, 60.21. MS (APCI) *m/z*: $[M - H]^+$ calcd for $C_{20}H_{20}N_2O_2S$, 351.12; found, 351.2. Anal. calcd for $C_{20}H_{20}N_2O_2S$: C 68.16, H 5.72, N 7.95; found: C 67.92, H 5.81, N 7.55.

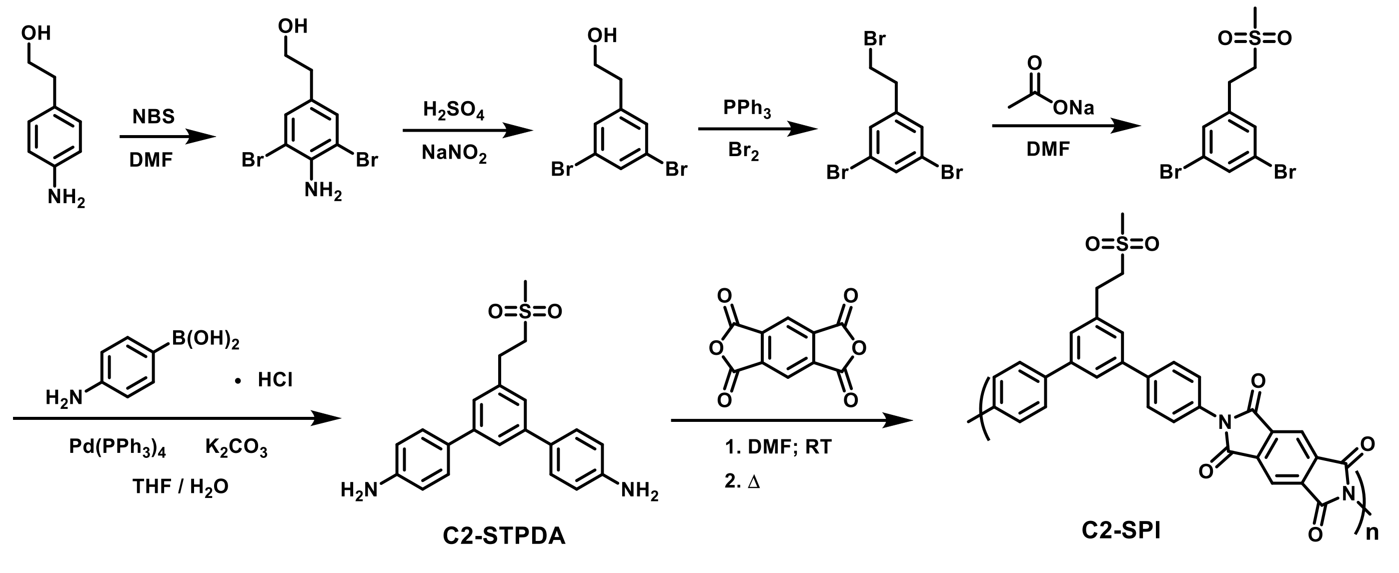

Scheme S3. Synthetic routes of the polyimide C2–SPI

Synthesis of 2-(4-amino-3,5-dibromophenyl)ethan-1-ol. To a stirred solution of 2-(4-aminophenyl)ethan-1-ol (20 g, 145.79 mmol) in DMF (140 mL) were slowly added NBS (51.90 g, 291.58 mol) at 0 °C, and the mixture was stirred at 0 °C for 3 h. Saturated brine was added to the reaction mixture. The resulting residue was filtered and dried to afford 4.02 g brown solid product with a yield of 93.5%. ^1H NMR (400 MHz, Chloroform-*d*) δ 7.29 (s, 2H), 5.11 (s, 2H), 3.53 (t, *J* = 6.7 Hz, 2H), 2.57 (t, *J* = 6.7 Hz, 2H). ^{13}C NMR (101 MHz, Chloroform-*d*) δ 135.75, 127.44, 125.31, 104.12, 58.69, 32.80. *m*/*z*: $[M]^+$ calcd for $C_8H_9Br_2NO$, 292.91; found 292.9. Anal. calcd for $C_8H_9Br_2NO$: C 32.58, H 3.08; found: C 33.35, H 3.21.

Synthesis of 2-(3,5-dibromophenyl)ethan-1-ol. 2-(4-amino-3,5-dibromophenyl) ethan-1-ol (20 g, 67.80 mmol), 15.3 mL concentrated H_2SO_4 and $NaNO_2$ (16.84 g, 244.09 mmol) were added into ethanol (250 mL), and the reaction mixture was heated to 80 °C for 6 h. Ethanol was removed by evaporation under reduced pressure, then water was added. The mixture was extracted with CH_2Cl_2 and evaporated under reduced pressure. The resulting mixture was purified by column chromatography using CH_2Cl_2 as mobile phase to afford 12.07 g white solid with a yield of 63.7%. ^1H NMR (400 MHz, Chloroform-*d*) δ 7.64 (d, *J* = 1.9 Hz, 1H), 7.48 (d, *J* = 1.8 Hz, 2H), 4.68 (t, *J* = 5.1 Hz, 1H), 3.61 (q, *J* = 6.2 Hz, 2H), 2.72 (t, *J* = 6.5 Hz, 2H). ^{13}C NMR (101 MHz, Chloroform-*d*) δ 132.17, 130.88, 122.96, 77.21, 62.96, 38.41. *m*/*z*: $[M]^+$ calcd for $C_8H_8Br_2O$, 277.89; found 277.9. Anal. calcd for $C_8H_8Br_2O$: C 34.32, H 2.88; found: C 35.01, H 2.67.

Synthesis of 1,3-dibromo-5-(2-bromoethyl)benzene. Triphenylphosphine (8.39 g, 31.98 mmol) was dissolved in CH_2Cl_2 (50 mL), and bromine (5.11 g, 31.98 mmol) was added dropwise in an ice bath and stirred at room temperature for 30 min. 2-(3,5-dibromophenyl)ethan-1-ol (7.46 g, 26.65 mmol) was added in an ice bath and stirred at room temperature for 1 h. The reaction mixture was evaporated under reduced pressure. The resulting residue was purified by column chromatography to afford 6.25 g colorless liquid with a yield of 68.4%. ^1H NMR (400 MHz, Chloroform-*d*) δ 7.70 (p, *J* = 2.3 Hz, 1H), 7.48 (d, *J* = 1.9 Hz, 2H), 3.76 (t, *J* = 7.0 Hz, 2H), 3.15 (t, *J* = 7.0 Hz, 2H). ^{13}C NMR (101 MHz, Chloroform-*d*) δ 144.29, 132.08, 131.36, 122.76, 37.57, 34.15. *m*/*z*: $[M]^+$ calcd for $C_8H_7Br_3$, 339.81; found 339.8. Anal. calcd for $C_8H_7Br_3$: C 28.03, H 2.06; found: C 28.45, H 2.33.

Synthesis of 1,3-dibromo-5-(2-(methylsulfonyl)ethyl)benzene. 1,3-dibromo-5-(2-bromoethyl)benzene. (3 g, 8.75 mmol) and sodium methanesulfinate (2.14 g, 21.00 mmol) were dissolved in DMF (25 mL) and reacted at 60 °C under argon for 3 h. The reaction mixture then poured into 1000ml of cold saturated NaCl aqueous solution, filtered and dried. Recrystallization in ethanol afforded 2.25 g white acicular crystal with a yield of 75.2%. ^1H NMR (400 MHz, Chloroform-*d*) δ 7.70 (t, *J* = 1.7 Hz, 1H), 7.61 (d, *J* = 1.8 Hz, 2H), 3.52 - 3.44 (m, 2H), 3.04 (dd, *J* = 7.2, 3.9 Hz, 2H). ^{13}C NMR (101 MHz, Chloroform-*d*) δ 144.31, 144.29, 132.08, 131.55, 131.36, 122.76, 37.57, 34.15. *m*/*z*: $[M]^+$ calcd for $C_9H_{10}Br_2O_2S$, 339.88; found 339.9. Anal. calcd for $C_9H_{10}Br_2O_2S$: C 31.60, H 2.95; found: C 32.1, H 3.21.

Synthesis of 5'-(2-(methylsulfonyl)ethyl)-[1,1':3',1''-terphenyl]-4,4''-diamine (C2-STPDA). 1,3-dibromo-5-(2-(methylsulfonyl)ethyl)benzene (2 g, 5.85 mmol) and $Pd(PPh_3)_4$ (0.22 g, 0.19 mmol) were dissolved in THF (50 mL). Then 3 M aqueous K_2CO_3 (16 mL), moderate Aliquat 336 and 4-aminophenylboronic acid hydrochloride (2.43 g, 22.93 mmol)

were successively added to the solution, followed by reaction at 70 °C under argon for 48 h. The mixture was allowed to cool down to room temperature. After collecting the organic layer by extraction with CH_2Cl_2 and evaporating the solvent, the product was purified by chromatography using CH_2Cl_2 and ethyl acetate as mobile phase to give 1.2 g white powder with a yield of 56.0%. ^{1}H NMR (400 MHz, Chloroform-*d*) δ 7.51 (t, *J* = 1.7 Hz, 1H), 7.47 – 7.40 (m, 4H), 7.38 (s,2H), 7.33 (d, *J* = 1.6 Hz, 2H), 6.68 – 6.62 (m, 4H), 5.22 (s, 4H), 3.56 - 3.48 (m, 2H), 3.11-3.03 (m, 2H), 3.01 (s, 3H). ^{13}C NMR (101 MHz, Chloroform-*d*) δ 148.88, 141.83, 139.45, 128.01, 127.83, 123.67, 121.22, 114.58, 55.08, 40.68, 28.57. *m*/*z*: $[M]^+$ calcd for $C_{21}H_{22}N_2O_2S$, 366.14; found 366.1. Anal. calcd for $C_{21}H_{22}N_2O_2S$: C 68.83, H 6.05; found: C 67.86, H 6.21.
